# Universalização da atenção primária à saúde: “coração” da reforma em saúde no Chile

**DOI:** 10.1590/0102-311XPT225624

**Published:** 2026-08-03

**Authors:** Patty Fidelis de Almeida, Márcia Cristina Rodrigues Fausto, Oscar Arteaga, Jose Zamorano, Manuel Navarrete, Lígia Giovanella

**Affiliations:** 1 Instituto de Saúde Coletiva, Universidade Federal Fluminense, Niterói, Brasil.; 2 Escola Nacional de Saúde Pública Sergio Arouca, Fundação Oswaldo Cruz, Fiocruz,Rio de Janeiro, Brasil.; 3 Facultad de Medicina, Universidad de Chile, Santiago, Chile.; 4 Servicio de Salud Metropolitano Occidente, Santiago, Chile.

**Keywords:** Atenção Primária à Saúde, Reforma dos Serviços de Saúde, Acesso Universal aos Serviços de Saúde, América Latina, Primary Health Care, Health Care Reform, Universal Access to Health Care Services, Latin America, Atención Primaria de Salud, Reforma de la Atención de Salud, Acceso Universal a los Servicios de Salud, América Latina

## Abstract

A proposta de universalização da atenção primária à saúde (APS) tornou-se a principal estratégia do governo chileno (2022-2026) de Reforma do Sistema de Saúde, sob os valores da equidade no acesso, qualidade dos cuidados e proteção financeira. O artigo identifica e analisa estratégias relacionadas à “universalização da APS” no que se refere à operacionalização, condução da reforma e medidas para ampliação e qualificação do acesso à APS. Foi realizado estudo de caso, com abordagem qualitativa, com base em entrevistas semiestruturadas com atores-chave, visitas in loco, complementadas por análise documental. A implementação da universalização da APS é apoiada por um Conselho Assessor e Comissões, nas quais se busca garantir o pluralismo político com vistas à governabilidade. Foi iniciada por meio de projetos pilotos, atualmente em 21 “Comunas Pioneiras”, cuja inscrição nos serviços de APS independe do tipo de asseguramento. Entre as estratégias para ampliar e qualificar o acesso à APS destacam-se: horário estendido de funcionamento dos centros de saúde; sistema de gestão remota da demanda; diretrizes para o manejo integral e integrado de condições crônicas com “atenção por duplas” e o “plano de cuidados consensuados”; “diálogos cidadãos” e cartografia dos ativos comunitários para fortalecer a participação social. No Chile, pós-derrota da proposta de Constituição em 2022 e em um contexto condicionado por conflitos políticos, baixa governabilidade, direcionalidade neoliberal das políticas públicas prévias, a universalização da APS emergiu como possível, consensual e sem resistências importantes, ganhando destaque frente à impossibilidade de reformas estruturais no sistema de saúde, que permanece dual.

## Abstract (en)

The proposal for the universalization of primary health care (PHC) has become the main strategy of the Chilean government (2022-2026) for reform of the healthcare system under the values of equity in access, quality of care, and financial protection. The article identifies and analyzes strategies related to the universalization of PHC with regards to operationalization, reform, and measures to expand and qualify access to PHC. A case study was carried out with a qualitative approach based on interviews with key actors during on-site visits complemented by documental analysis. The implementation of the universalization of PHC is supported by an advisory council and commissions that seek to ensure political pluralism with a view to governability. It was initiated through pilot projects currently in 21 “pioneer communes”. The following strategies to expand and qualify access to PHC stand out: extended office hours at health centers; remote demand management system; guidelines for the comprehensive management of chronic conditions with “care by pairs” and a “consensual care plan”; “citizen dialogues” and mapping of community assets to strengthen social participation. After the defeat of the proposed Constitution in 2022 and in a context conditioned by political conflicts, low governability, and the neoliberal directives of previous public policies in Chile, the universalization of PHC emerged as possible, consensual, and without substantial resistance, gaining prominence in the face of the impossibility of structural reforms in the healthcare system, which remains polarized.

## Resumen

La propuesta de universalización de la atención primaria de salud (APS) se ha convertido en la principal estrategia del Gobierno chileno (2022-2026) para la reforma del sistema sanitario, bajo los valores de equidad en el acceso, calidad de la atención y protección financiera. Este artículo identifica y analiza estrategias relacionadas con la “universalización de la APS” en lo que se refiere a la operatividad, la conducción de la reforma y las medidas para ampliar y mejorar el acceso a la APS. Se realizó un estudio de caso, con un enfoque cualitativo, basado en entrevistas semiestructuradas con actores clave, visitas presenciales, complementadas con análisis documental. La implementación de la universalización de la APS cuenta con el apoyo de un Consejo Asesor y de Comisiones, con el fin de garantizar el pluralismo político con vistas a la gobernabilidad. Se inició mediante proyectos piloto, actualmente en 21 “Comunas Pioneras”, cuya inscripción en los servicios de APS es independiente del tipo de seguro. Entre las estrategias para ampliar y mejorar el acceso a la APS destacan: horario ampliado de funcionamiento de los centros de salud; sistema de gestión remota de la demanda; directrices para el manejo integral e integrado de las afecciones crónicas con “atención por parejas” y el “plan de cuidados consensuados”; “diálogos ciudadanos” y cartografía de los activos comunitarios para fortalecer la participación social. En Chile, tras el rechazo de la propuesta de Constitución en 2022 y en un contexto marcado por los conflictos políticos, la baja gobernabilidad y la orientación neoliberal de las políticas públicas anteriores, la universalización de la APS surgió como una posibilidad consensuada y sin resistencias importantes, ganando protagonismo ante la imposibilidad de llevar a cabo reformas estructurales en el sistema de salud, que sigue siendo dual.

## Introdução

A Constituição chilena de 1980, promulgada durante a ditadura, não reconhece a saúde como um direito de cidadania, cabendo ao Estado garantir acesso igualitário às ações e serviços de saúde, enquanto o cidadão pode eleger a cobertura no sistema público ou privado. Com a posse do governo de Gabriel Boric (2022-2026), no ano de 2022, foi apresentada proposta constitucional em resposta às mobilizações de 2019, que ampliava direitos sociais por meio de reformas estruturais para o enfrentamento das profundas desigualdades ^1^. Para a saúde, propugnou-se a criação de um Sistema Nacional de Saúde universal, público e integrado, regido pelos princípios da solidariedade. No plebiscito nacional, realizado em 2022, a proposta foi derrotada por ampla maioria. Na mesma direção, o veto à realização da reforma tributária, pela Câmara dos Deputados, em 2023, encerrou as possibilidades de acordo político para um financiamento capaz de sustentar políticas públicas mais inclusivas e equitativas ^2^.

Assim, a estrutura do sistema de saúde chileno permanece dual e segmentada. Embora o país represente um caso exemplar do poder dos interesses privados e empresariais no setor saúde, que obstaculizam reformas estruturais, a criação do Serviço Nacional de Saúde, em 1952, e as ações implementadas até a sua interrupção em 1973 conformam um legado institucional público, com ampla rede de serviços estatais ^3^. Em que pese a manutenção da segmentação da cobertura, o seguro público − Fundo Nacional de Saúde (Fondo Nacional de Salud, Fonasa) − era responsável pela cobertura de 84% da população em setembro de 2024, e o seguro privado, integrado por Instituições de Saúde Previdenciária (Instituciones de Salud Previsional, Isapre), pela cobertura de 16%, no mesmo ano ^4^. Ademais, o sistema de saúde chileno, principalmente pela priorização da atenção primária à saúde (APS) no subsetor público, alcançou resultados expressivos no controle de doenças transmissíveis e melhoria da qualidade de vida, posicionando o país entre os melhores resultados em saúde na América Latina ^5^. Destaca-se também a capacidade do subsistema público responder às emergências e desastres naturais e sanitários, a exemplo da atuação durante a pandemia de COVID-19 ^6^.

Nesse contexto, a proposta da “APS universal” ou “universalização da APS” é assumida como uma das etapas em direção a um sistema universal de saúde - o “coração” da Reforma ^7^. Durante o ano de 2023, o Ministério da Saúde (Minsal) iniciou a implementação progressiva da universalização da APS, por meio de sete projetos pilotos, em municípios selecionados nas denominadas - “Comunas Pioneiras”. De todo modo, a universalização da APS foi precedida por um conjunto de reformas incrementais orientadas ora à ampliação do acesso às ações e serviços de saúde, ora ao modelo assistencial e prestação dos serviços de APS, durante o processo de democratização do país a partir dos anos 1990 ^3^. Em 2005, o Regime de Garantias Explícitas em Saúde (GES) passou a assegurar oportunidade de acesso, qualidade e proteção financeira para agravos específicos, ampliados ao longo do tempo, até alcançar 87 patologias no ano de 2024 (jun/2024) ^8^. Outra reforma essencial da APS começou em 2005 a partir da implementação do Modelo de Assistência Integral em Saúde da Família e da Comunidade (MAIS) operado por meio da transformação dos consultórios e centros de saúde tradicionais em centros de saúde familiares (CESFAM) e em centros de saúde comunitários (CECOSF). O MAIS buscou ampliar a concepção de modelo assistencial - centrado nas pessoas, capaz de prover cuidados integrais e garantir continuidade - por meio da atuação multiprofissional em redes de atenção à saúde, intersetorial e com participação social ^9^.

Tais princípios são reafirmados pela universalização da APS, frente ao alcance incompleto do MAIS em eixos centrais como a colaboração interprofissional e os cuidados centrados na pessoa ^10^, a despeito do aumento expressivo do número de CESFAM. Assim, a universalização da APS se apresenta como uma “janela de oportunidades” para universalizar a APS e provocar mudanças na organização do sistema de saúde e do modelo assistencial, com base em uma concepção abrangente que alie ampliação do acesso e enfoque territorial, familiar, comunitário, com trato digno e respeitoso aos usuários ^2^.

Este artigo tem como objetivo identificar e analisar estratégias relacionadas à “universalização da APS”, eixo estruturante da Reforma de Saúde no Chile, no que se refere à operacionalização, condução e medidas para ampliação e qualificação do acesso. Busca-se identificar facilitadores, resistências, apoios e, sobretudo, lições aprendidas que possam informar políticas nacionais e internacionais de fortalecimento da APS como pilar estruturante dos sistemas de saúde. 

## Metodologia

Trata-se de estudo de caso, com abordagem qualitativa, tendo como principal fonte de informação entrevistas semiestruturadas com informantes-chave, complementadas por análise documental e visitas de campo a serviços de APS que fazem parte dos projetos piloto da universalização da APS em três Comunas Pioneiras.

No estudo, buscou-se contemplar atores do nível macro de condução da reforma, responsáveis por decisões políticas acerca dos direitos, financiamento e macrorregulação do sistema de saúde; do nível meso ou de gestão a cargo da implementação dos mecanismos operativos que dão suporte às ações planejadas; e do nível micro, no qual se efetiva a oferta dos cuidados em saúde ^11^. A identificação e a análise dos principais componentes estratégicos relacionados à operacionalização, condução da reforma e medidas para ampliar e qualificar o acesso no âmbito da “universalização da APS” foram guiadas por dimensões e categorias selecionadas com base no marco para o monitoramento da APS da Organização Mundial da Saúde ^12^, cotejado e adaptado à matriz de análise baseada em uma abordagem integral e integrada da APS desenvolvida no âmbito do Instituto Sul-Americano de Governo em Saúde (ISAGS) da União das Nações Sul-Americanas (UNASUL) ^13^ ^,^ ^14^.

Assim, foram realizadas entrevistas com atores sociais com papel importante na condução da universalização da APS no Ministério da Saúde e em conselhos assessores, incluindo representantes de organizações nacionais de profissionais e de usuários. No nível meso, privilegiou-se os gestores dos *Servicios de Salud*, estrutura regionalizada de gestão do sistema público; gestores municipais e responsáveis territoriais pela condução da universalização da APS em três experiências locais e em uma regional. Para compreender a experiência e percepção dos envolvidos na concretização da reforma nos territórios, no nível local, foram realizadas entrevistas coletivas com profissionais e gerentes de serviços de APS, complementadas por visitas in loco, em agosto de 2024. Adicionalmente, foram entrevistados representantes da academia envolvidos em estudos e reflexões críticas acerca do sistema de saúde chileno. O Quadro 1 apresenta os participantes da pesquisa, conforme nível e função, de forma genérica para minimizar a identificação.

**Quadro 1:** Informantes-chave entrevistados. Chile, 2024.

NÍVEL	FUNÇÃO	N
Macro: Ministério da Saúde; Comissões assessoras; organizações nacionais de profissionais e de usuários	Comissão Saúde Senado - representante da Academia	1
	Minsal − condução da universalização da APS	3
	Conselho Assessor da Reforma	1
	Conselho Assessor da Reforma representante de usuários - ANCOSALUD	1
	Fonasa/Academia	1
	Entidade de representante de profissionais da APS - CONFUSAM	1
Meso: Serviços Regionais de Saúde; gestão em instâncias municipais	Gestão Serviços Regionais de Saúde	4
	Gestão/gerência saúde municipal	4
	Referente a universalização da APS	3
Micro: serviços de APS e territoriais; profissionais; representante sociedade civil	Diretoria CESFAM	3
	Profissionais de saúde e de outros serviços territoriais	11
	Comissão de Saúde Municipal − usuário	1
Academia	Pesquisadores/Professores	10
Total		44

ANCOSALUD: Associação Nacional de Conselhos de Usuários da Saúde Pública; APS: atenção primária à saúde; CESFAM: Centro de Saúde Familiar; CONFUSAM: Confederação Nacional de Funcionários e Funcionárias da Saúde Municipal; Fonasa: Fundo Nacional de Saúde; Minsal: Ministério da Saúde.

Fonte: elaboração própria com base nas entrevistas e visitas.

As percepções e experiências dos atores estratégicos foram analisadas de forma imbricada, de modo a proporcionar um panorama amplo dos objetivos, conteúdo e estratégias de implementação da universalização da APS. Para a produção dos resultados, procedeu-se à análise de conteúdo temática de todo o material com suas respectivas etapas de categorização, descrição e interpretação. A descrição dos resultados foi realizada, em uma perspectiva indutiva, com base nas entrevistas e dados das observações realizadas durante as visitas. De forma complementar foram consultados documentos, alguns de circulação restrita disponibilizados durante a visita, que dispõem sobre os principais marcos e alguns resultados da universalização da APS.

Os resultados são apresentados com base nas dimensões que compõem o marco teórico conceitual (Quadro 2) e, em uma perspectiva crítica, valendo-se das experiências dos participantes da pesquisa. Buscou-se garantir a qualidade e a validade dos achados triangulando-se as informações da análise documental, observações *in loco* e, sobretudo, a percepção dos diferentes grupos de informantes-chave.

**Quadro 2:** Marco conceitual para análise de componentes estratégicos da atenção primária à saúde (APS). Chile, 2025.

DIMENSÕES	DEFINIÇÃO	CATEGORIAS
Compromisso político, liderança e governança	Compromisso político e liderança que posicionem a APS no centro dos esforços para alcançar a cobertura universal de saúde e reconheçam sua ampla contribuição para o alcance dos Objetivos de Desenvolvimento Sustentável (ODS). Envolve estruturas de governança, políticas e regulação que apoiem a APS ao interior do setor saúde e demais setores das políticas públicas, com participação social	Proteção social em saúde e segmentos de cobertura
		Existência de legislação acerca do direito à saúde
		Existência de política nacional de saúde orientada pela APS e ao acesso universal
Financiamento e alocação de recursos	Financiamento adequado para a APS, de forma a promover equidade no acesso e viabilizar cuidados e serviços de alta qualidade.	Financiamento da APS
Modelo de atenção	Modelos de atenção que promovam a APS de alta qualidade, centrada nas pessoas e funções essenciais de saúde pública.	Abordagens e modelos de atenção na APS
		Infraestrutura física
		Coordenação dos cuidados e integração nas redes de atenção à saúde
		Força de trabalho para a APS
		Uso de tecnologias digitais em saúde na APS
Engajamento da comunidade e de outros atores	Participação das comunidades e outros atores para identificar problemas e soluções, e priorizar ações por meio do diálogo intersetorial, considerando políticas e ações interculturais.	Estratégias nacionais, subnacionais e locais para a participação comunitária
		Mecanismos de coordenação entre setores com participação e compromisso comunitário

Fonte: elaboração própria com base em Organização Mundial da Saúde & Fundo das Nações Unidas para a Infância ^12^; Giovanella et al. ^13^, Almeida et al. ^14^.

O estudo foi aprovado pelo Comitê de Ética em Pesquisa da Escola Nacional de Saúde Pública Sergio Arouca, Fundação Oswaldo Cruz (parecer nº 6.800.007).

## Resultados 

### Compromisso político, liderança e governança: a arquitetura da universalização da APS

Com a derrota do plebiscito sobre a nova proposta constitucional, em 2022, o ímpeto e as possibilidades políticas para transformações estruturais no sistema de proteção social chileno diminuíram. No campo da saúde, a proposta da universalização da APS emergiu como possível, consensual e sem resistências expressivas, na avaliação de todos os grupos de participantes, ganhando maior importância frente à impossibilidade de reformas estruturais. Entrevistados do nível macro consideraram que, embora seja uma reforma incremental, é parte de um aprendizado institucional contínuo e de valorização do sistema de saúde público, enquanto se busca avançar em mudanças estruturais.

O objetivo central da universalização da APS ancorou-se na ampliação da cobertura dos serviços públicos e gratuitos de APS a todas os chilenos, independentemente da afiliação ao subsistema público ou privado. Para contextualizar a proposta de Reforma, o Quadro 3 sintetiza aspectos da proteção social em saúde no país e sua relação com a universalização da APS.

**Quadro 3:** Proteção social em saúde e segmentos de cobertura. Chile, 2024.

Proteção social em saúde baseada em seguros, em regime dual, segmentado em dois principais subsetores de afiliação: Seguradoras privadas - instituições de saúde previdenciárias (Isapre) - asseguram trabalhadores do mercado formal de maior renda e cobram prêmios adicionais às contribuições obrigatórias para acesso a planos diferenciados - cobre 16% da população (2024) Seguradora pública - Fundo Nacional de Saúde (Fonasa) - filia trabalhadores do mercado formal por meio de contribuições apenas do trabalhador (7% do salário mensal), trabalhadores independentes e grupos populacionais de baixa renda não inseridos no mercado formal na modalidade de assistência Institucional (MAI) com financiamento público - cobre 84% da população (2024) Segurados do Fonasa podem escolher uma modalidade de livre eleição (MLE) para acesso a prestadores privados por meio de *voucher* e copagamentos O cuidado na APS é totalmente gratuito, com abolição pelo governo Bóric de todos os copagamentos (*Copago Cero*). Nos serviços privados permanecem os copagamentos. O acesso é restrito aos beneficiários do Fonasa, que, voluntariamente, registram-se nos centros de saúde, sob responsabilidade municipal Mudanças a partir da universalização da APS Objetiva garantir a cobertura universal de APS, independentemente do seguro de saúde, com vistas a garantir a efetivação dos cuidados previstos no Modelo de Assistência Integral em Saúde da Família e da Comunidade (MAIS) - centrado nas pessoas, com ampla equipe multiprofissional, ação intersetorial e participação social Nos projetos piloto, a universalização da APS incentiva medidas ativas para a inscrição dos segurados das Forças Armadas e Policiais (que têm um sistema próprio), segurados Fonasa da modalidade de livre eleição ainda não estão registrados nos centros de saúde familiares (CESFAM) ou centros de saúde comunitários (CESCOF), garantindo a todos o acesso ao cuidado integral e gratuito na APS

APS: atenção primária à saúde.

Fonte: Consejo Asesor APS Universal ^15^; Secretaría General de la Presidencia ^4^.

Quanto à governança, para a implementação da universalização da APS foram estabelecidos um Conselho Assessor e Comissões, cujas representações buscavam assegurar o pluralismo político com vistas à viabilidade e ampliação da base de sustentação da proposta. No ano de 2022, a Ministra da Saúde, principal liderança da universalização da APS, instituiu o Conselho Assessor da Atenção Primária Universal (*Consejo Asesor de la Atención Primaria Universal*, Quadro 4), que contava com atores históricos como ex-ministros da saúde dos campos progressistas e da direita, além dos grêmios profissionais e representações dos usuários. A proposta da universalização da APS recebeu apoio, inclusive, da corporação médica e de prefeitos de diferentes matizes ideológicas e partidos políticos, considerados atores muito relevantes, conforme a avaliação dos entrevistados dos níveis macro e meso. Assim, a universalização da APS pôde ser iniciada, a despeito dos constrangimentos políticos e financeiros às reformas estruturais. No ano de 2023, no âmbito do Conselho Assessor foram formadas cinco mesas temáticas para realizar diagnósticos e proposições para o avanço da universalização ^15^ (Quadro 4). A Comissão de Saúde do Senado, constituída em 2024, e equipes “motoras” locais com representações do Minsal, Comunas e estabelecimentos de saúde responsáveis pela gestão das mudanças nos territórios também fazem parte da estrutura de governança da universalização da APS (Quadro 4).

**Quadro 4:** Governança da universalização da atenção primária à saúde (APS). Chile, 2024.

AÇÕES E ESTRATÉGIAS	DESCRIÇÃO E ANÁLISE
Características gerais	População em 2024: 19.658,839 29 Serviços de saúde - correspondem aos territórios regionais, nos quais se articulam hospitais e centros de saúde especializados que prestam serviços na rede pública Comunas (municípios) responsáveis pela APS - 346 Número de estabelecimentos de APS (2024):
	Centros de Saúde Familiar (CESFAM) - 621 Centros Comunitários de Saúde Familiar (CECOSF) - 291 Postos de Saúde Rurais - 1.118 Hospitais Comunitários de baixa complexidade - 100 Serviços de Atenção Primária de Urgência (SAPU) - 234 Serviços de Urgência Rurais (SUR) - 166 Serviços de atenção primária de urgência de alta resolutividade (SAR) - 93
Comunas Pioneiras - 7 em 2023 e mais 14 em 2024	2023: Canela, Alhué, Renca, La Cruz, Coltauco, Linares e Perquenco; 2024: Arica, Alto Hospicio, Tocopilla, Caldera, Illapel, Quilpué, Chillán Viejo, Curanilahue, Angol, Valdivia, Puerto Varas, Aysén, Puerto Natales y La Pintana Avaliações:
	Comunas Pioneiras eleitas entre municípios com distintas experiências e tipos de territórios (rural e urbano, por exemplo), pertencentes a diferentes serviços de saúde e espectros políticos, sobretudo para se garantir uma base de apoio Entrevistados representantes dos profissionais dos níveis meso, micro e academia sinalizaram acordos políticos entre Minsal e prefeitos para eleição das Comunas Pioneiras, inclusive em algumas que não possuíam o modelo MAIS
*Consejo Asesor de la Atención Primaria Universal*	Função: assessorar o governo na implementação da universalização da APS, apresentando diagnósticos, proposições e acompanhamento dos resultados. O Conselho é composto por todos os ex-Ministros da Saúde; parlamentares; governadores regionais; usuários; sindicatos e grêmios; colégios profissionais; instituições científicas; organismos internacionais Mesas temáticas propostas: modelo de atenção, participação, intersetorialidade e promoção da saúde, resolutividade, formação de pessoal e financiamento
*Comisión Técnica Asesora para la Comisión de Salud del Senado*	Tem como função elaborar recomendações que contribuam para os projetos de lei que o governo deveria apresentar em outubro de 2024. Estava conformada por 5 especialistas apoiadores do atual governo e 05 da oposição, indicados pelo Senado. No momento de realização do campo (agosto/2024), a Comissão estava em sua terceira composição, cujos membros eram convocados pela Comissão de Saúde do Senado
Equipes “motoras”	Equipe/profissionais designados ou contratados com recursos previstos pela universalização da APS para liderar e assessorar a implementação das ações previstas. São compostas por lideranças atuantes no Minsal e nos espaços loco-regionais para orientar as mudanças necessárias nas Comunas e nos serviços de saúde indispensáveis à implementação da universalização da APS

MAIS: Modelo de Assistência Integral em Saúde da Família e da Comunidade; Minsal: Ministério da Saúde;

Fonte: elaboração própria com base em informações do Ministério da Saúde, entrevistas com informantes-chave e outras fontes documentais e bibliográficas.

A reforma começou gradualmente, de forma a diminuir resistências, por meio de sete projetos piloto, a partir de 2023, e, até agosto de 2024 estava presente em 21 Comunas, avançando lentamente, conforme avaliação dos quatro grupos de entrevistados. A despeito dos critérios técnicos, foram mencionados acordos políticos para eleição das Comunas Pioneiras (Quadro 4), mencionado por parte dos atores do nível meso.

Um aspecto importante da universalização da APS e de dissenso nas avaliações relacionou-se à implementação das medidas e eixos estratégicos sem um marco legislativo, ou seja, sem a criação de leis ou decretos. Se por um prisma, a negociação no parlamento condicionaria um conjunto de mudanças nem sempre adequadas do ponto de vista técnico, assistencial e dos resultados esperados, alguns entrevistados, sobretudo do nível meso e da academia, destacaram que a ausência de um marco legal facilitaria o desmonte e a descontinuidade da Reforma.

### Financiamento e modelo de atenção

Em relação à APS, prevaleceu a percepção de que “faz muito sentido atender e cuidar de todas as pessoas de um território” (entrevistado nível macro); “APS como um bem público” (entrevistada academia) - não se constituindo uma bandeira exclusiva de determinado espectro político, avaliação respaldada por participantes de todos os grupos. Destacou-se que o segmento público de saúde oferecia cobertura a aproximadamente 80% da população por meio de um “potente sistema de saúde pública” (entrevistado nível macro).

A proposta da universalização da APS ancorava-se em eixos e ações estratégicos como a ampliação e a oportunidade de acesso (acesso cercano), diálogos cidadãos, efetividade e qualidade, ativos comunitários para a promoção da saúde, componentes afinados a um modelo de atenção integral de APS (Quadro 2). A base das ações era composta pelo plano de saúde familiar que incorporava os programas de saúde do Minsal.

No país, a APS era financiada principalmente por valores *per capita* e pelo Programa de Reforçamento da APS (*Programa de Reforzamiento de la Atención Primaria de Salud*, PRAPS), recursos repassados pelo Minsal, além de recursos provenientes dos municípios ^16^. Os recursos para a implementação da universalização da APS representaram aportes adicionais via PRAPS, que incluíram um empréstimo do Banco Mundial, condicionado a um marco avaliativo. As avaliações, bastante consensuais entre o conjunto dos participantes, destacaram a garantia de estabilidade e a ampliação do financiamento como um dos principais desafios à sustentabilidade e ampliação da universalização da APS (Quadro 5).

**Quadro 5:** Eixos e ações estratégicas da implementação da universalização da atenção primária à saúde (APS): modelo de atenção e financiamento. Chile, 2024.

AÇÕES E EIXOS ESTRATÉGICOS	DESCRIÇÃO E ANÁLISE
Modelo de Atenção − eixos centrais da universalização da APS	Acesso *cercano*: inscrição universal; modernização do agendamento de procedimentos nos serviços de APS; diálogos cidadãos; horários estendidos de atendimento; pontos de atenção mais próximos dos territórios Efetividade: cuidados integrais consensuados, mapeamento dos recursos comunitários, capacitação dos profissionais da saúde; integração à rede de serviços de saúde; novo plano de saúde e incorporação de tecnologias de informação e comunicação Qualidade: redesenho do financiamento; otimização dos processos de gestão; investimentos em infraestrutura e dados; modelo de qualidade
Financiamento da APS pelo Minsal e mudanças a partir da universalização da APS	***Per capita*:** valor fixo por cada pessoa inscrita em estabelecimentos Fonasa transferido pelo Minsal aos municípios. O valor é condicionado à inscrição dos usuários no centro de saúde, indexado com base em variáveis como pobreza municipal (reconhecendo comunas pobres e não pobres), ruralidade e algumas variáveis demográfico-epidemiológicas (% de população idosa). O montante *per capita* representa 64,1% do financiamento total da APS. Nas Comunas com a universalização da APS, segurados Isapre e das Forças Armadas inscritos são acrescidos ao financiamento *per capita*. A exceção são 64 municípios com menos de 3.500 habitantes que recebem um valor fixo, uma vez que os custos para provisão dos serviços de saúde não são compatíveis ao financiamento *per capita* **Programas de Reforçamento da APS** (PRAPS): repasses nacionais que financiam prestações ou ações específicas na APS e complementam o financiamento *per capita*, a partir de um valor prospectivo. Representam 29,1% do financiamento da APS. Com a universalização da APS, foram incorporadas novas prestações **Recursos municipais**: aportes municipais, não obrigatórios, destinados à APS, dependem da capacidade socioeconômica local. Representam 6,8% do total de recursos da APS. Em alguns pilotos da APS-U houve aporte importante de recursos municipais Avaliações: Principal desafio para sustentabilidade da reforma. No interior do governo nacional, maiores esforços de convencimento foram direcionados ao Ministério da Fazenda, fundamental para a garantia dos recursos para manutenção e expansão da universalização da APS. Uma das estratégias foi a apresentação, aos ministros da área econômica, de indicadores de processo e resultados positivos em saúde alcançados por meio da APS Empréstimo do Banco Mundial para financiamento da universalização da APS considerado crítico quanto à sustentabilidade, uma vez que a duração seria de 4 anos, além da disponibilidade de recursos nacionais para a expansão às demais Comunas. Contudo, o aporte de novos recursos via PRAPS e os valores *per capita* com aumento das inscrições foi considerado um elemento central para garantir a implementação da universalização da APS Principal utilização dos recursos da universalização da APS − melhoria da infraestrutura dos equipamentos de saúde, contratação de profissionais para a extensão do funcionamento das unidades, realização dos diagnósticos comunitários, entre outros; Críticas do conjunto dos entrevistados direcionadas à insuficiência de recursos para se avançar com a universalização da APS com maior rapidez e garantia de equidade
Iniciativas do Programa de Reforçamento da APS em vigor em 2024	Apoio à gestão dos *serviços de saúde*; apoio à gestão local; boas práticas; espaços amigáveis para adolescentes; fundos para farmácia; fortalecimento dos recursos humanos em APS; migrantes; prestações odontológicas GES; odontologia integral; saúde escolar; reabilitação com base comunitária; saúde rural; Serviço Nacional de Menores (SENAME); Serviço de Atenção Primária de Urgência (SAPU); Serviço de Atenção Primária de Urgência de Alta Resolução (SAR); Serviço de Atenção Primária de Urgência de Alta Resolutividade (SUR); programa Vida Saudável; Estratégias de reforço em APS para enfrentar a pandemia de COVID-19; cuidados paliativos universais; continuidade dos cuidados preventivos; TEA (atenção integral ao desenvolvimento infantoadolescente); idosos autônomos; Centros de Salud Comunitarios (CECOSF); Chile cresce contigo; demência; capacitação e formação; Modelo de Assistência Integral em Saúde da família e da comunidade (MAIS); Programa de Formação de Especialistas em APS (FENAPS); fortalecimento das capacidades; imagens diagnósticas; manutenção da infraestrutura APS; melhoria do acesso à atenção odontológica; programas ministeriais; missões de formação e aperfeiçoamento em outros países/no estrangeiro); resolutividade na APS; saúde mental; programa *sembrando sonrisas*; teleeletrocardiograma; detecção, intervenção e referência assistida ao álcool, tabaco e outras drogas; universalização da APS; programa de acompanhamento em saúde mental infantil (PASMI); Programa Especial de Saúde dos Povos Indígenas (PESPI)

Fonasa: Fundo Nacional de Saúde; GES: Regime de Garantia Explícitas de Saúde; Minsal: Ministério da Saúde.

Fonte: elaboração própria com base em informações do Ministério da Saúde, entrevistas com informantes-chave e outras fontes documentais e bibliográficas.

A seguir, são apresentadas componentes do modelo de atenção da universalização da APS, destacando-se as principais medidas relacionadas à qualificação do cuidado e ampliação do acesso implementadas em municípios piloto e as avaliações do processo pelos atores.

### Inscrição universal à APS

Um eixo central da universalização da APS foi a possibilidade que qualquer pessoa que residisse ou trabalhasse nos territórios piloto pudesse se inscrever em estabelecimentos de APS da Comuna, independentemente do tipo de asseguramento. Os recursos *per capita* para os novos inscritos eram repassados aos municípios pelo Minsal. Na avaliação dos entrevistados dos níveis macro e meso, embora tenha sido ampliada a possibilidade de acesso aos serviços públicos de APS, não houve incremento importante de segurados privados e das Forças Armadas. O aumento mais expressivo ocorreu entre os usuários Fonasa. Observou-se, em algumas regiões do país, o incremento da inscrição de imigrantes, sobretudo venezuelanos. No ano de 2023, entre as sete Comunas Pioneiras, dos 49.338 potenciais novos usuários, 82,3% foram inscritos, sendo 84,4% Fonasa, 14,6% Isapres e 1% das Forças Armadas ^17^.

A maior parte dos entrevistados de todos os grupos considerou que os serviços de APS funcionavam, eram bem organizados e com grande capilaridade, inclusive em áreas remotas. Ademais, nos últimos anos houve investimentos na infraestrutura dos estabelecimentos, o que colaborou para a percepção positiva. Uma das entrevistadas da academia destacou a importância da APS pública durante a pandemia.

Diversas ações foram desenvolvidas pelas equipes de APS para incremento das inscrições como campanhas comunicacionais e ações em espaços públicos. Em uma das Comunas visitadas - La Cruz - com recursos da universalização da APS foi contratada uma jornalista encarregada pela comunicação com a população, manejo das redes sociais e rádios comunitárias, medida considerada fundamental para o êxito das mudanças propostas - “como a política pública se comunica com a população” (entrevistado nível micro). Entre os segurados privados havia receio quanto à perda do asseguramento, caso se inscrevessem no CESFAM, o que também foi objeto das campanhas informativas.

Houve divergência na avaliação dos atores quanto à inscrição universal. A maior parte considerou a ação relevante para o aumento do valor da APS e do sistema público pelo conjunto da população. No sentido oposto, entrevistados da academia argumentaram que a medida poderia ampliar as iniquidades, uma vez que o sistema público não possuiria a infraestrutura para atender ao incremento da população segurada pelas Isapres. De toda forma, foi destacado que a pandemia de COVID-19 havia borrado as fronteiras entre os sistemas público e privado, ampliando a valoração positiva dos serviços públicos. A capilaridade dos estabelecimentos foi mencionada como um fator de estímulo e satisfação dos usuários Isapre e Forças Armadas, diminuindo barreiras geográficas de acesso. Além disso, a adesão de segurados para inscrição na universalização da APS foi favorecida pela crise das Isapres, em decorrência de sentenças judiciais que poderiam levá-las ao colapso financeiro. O Supremo Tribunal, em decisão emitida em 2022, obrigara as Isapres a ajustar o valor dos seus planos e a devolver retroativamente aos seus segurados os prêmios cobrados em excesso pela não aplicação da Tabela Única de Fatores de Risco, desde 2020 ^18^. Após mais de um ano de debates, foi promulgada em maio de 2024 a Ley Corta de las Isapres ^19^, que ampliou os prazos para a devolução dos recursos aos segurados. Em meio às incertezas, segurados Isapre aderiram à universalização da APS.

### Horários estendidos e manejo remoto da demanda 

Soma-se à estratégia de inscrição universal, medidas para ampliação e qualificação do acesso aos estabelecimentos de APS por meio de duas ações principais. A primeira foi a extensão do horário de funcionamento dos Centros de Saúde - de 8:00 às 17:00 - para de 8:00 às 20:00 e sábados, de 9:00 às 13:00 horas. A medida foi viabilizada por financiamento específico para o pagamento adicional de 30 horas/profissional. Em geral, as equipes básicas dos CESFAM eram compostas por médicos, enfermeiros, matronas (profissionais de saúde que realizam atenção integral à saúde materno-infantil), odontólogos, psicólogos, assistentes sociais, fisioterapeutas, nutricionistas, fonoaudiólogos e terapeutas ocupacionais (como algumas variações entre os serviços) e técnicos de nível superior - chamados TENS. A ampliação do funcionamento poderia ser operacionalizada com a equipe completa ou parte dos profissionais. Entre as Comunas Piloto, todos os estabelecimentos haviam cumprido a meta de extensão horária ^16^, com avaliações positivas quanto à ampliação do acesso. Todavia, nesse ponto, se concentrou parte importante das críticas à universalização da APS, enfatizada pela representação dos profissionais. Uma das razões seria a insuficiência de horas/profissionais, em especial, de médicos, para novas contratações, a despeito da disponibilidade dos recursos financeiros. Dessa forma, a extensão horária era cumprida pelas próprias equipes em atuação no horário regular, provocando importante sobrecarga laboral.

Outra ação considerada estratégica para a redução de barreiras e oportunidades de acesso foi a implantação de um sistema de gestão remota da demanda, denominado Telessaúde, plataforma desenvolvida pelo Minsal, disponível para Comunas com e sem universalização da APS. Por meio do sistema online, os usuários poderiam preencher um formulário com os contatos pessoais e descrição de sua demanda. Além do gerenciamento remoto da demanda, a plataforma do Telessaúde possuía três componentes − teleeducação, telemedicina e teleassistência.

Cada estabelecimento de APS definiu a melhor estratégia para o gerenciamento da demanda remota. Em um CESFAM visitado, na Comuna de Renca, um médico e uma matrona possuíam turnos protegidos para análise das demandas, respostas e contato com os usuários via telefone. Pedidos de agendamento de consultas, atestados médicos, renovação de receitas estavam entre as solicitações possíveis e mais comuns via sistema. Muitas demandas eram resolvidas sem necessidade de ida do usuário ao serviço.

A implantação da estratégia enfrentou dificuldades relacionadas à conectividade, reorganização do processo de trabalho para atender às demandas remotas, além de medidas de comunicação e mobilização da população. Contudo, a estratégia foi considerada um aspecto bastante positivo para a ampliação do acesso e resolutividade da APS, destacada, principalmente, por entrevistados dos níveis meso e micro.

### Estratégia de Cuidado Integral Centrado nas Pessoas

No ano de 2021, o Minsal publicou o marco conceitual da Estrategia de Cuidado Integral Centrado nas Pessoas (ECICEP), protocolo que orientava o desenvolvimento das ações de promoção, prevenção e manejo de usuários com multimorbidade, tendo como base princípios do MAIS ^20^, experiências exitosas de cuidados integrais no país e os marcos teóricos e operativos da Organização Pan-Americana da Saúde (OPAS), conforme destacou o representante do Minsal.

O protocolo ECICEP antecedia a universalização da APS e poderia ser adotado em estabelecimentos de saúde que não faziam parte dos pilotos. O objetivo era superar a fragmentação do atendimento por patologias - “atenções programáticas” - que prevaleciam na APS chilena. Em 2023, a estratégia foi incorporada como um dos componentes da universalização da APS, com destinação de recursos via PRAPS, embora a implementação fosse bastante heterogênea e incipiente nas Comunas Piloto visitadas.

Em linhas gerais, por meio da estratificação de risco, diferentes estratégias para oferta de cuidados poderiam ser adotadas, entre as quais os “planos de cuidados consensuados” com os usuários e o “atendimento por duplas” de profissionais, compostas por médico, preferencialmente médico de família e outro profissional, a ser definido conforme as necessidades de cada pessoa ou vínculos previamente estabelecidos. O ingresso na ECICEP previa a construção de um plano de cuidado integral consensuado, no qual eram acordados objetivos de curto e longo prazo, buscando melhorar o autocuidado e o bem-estar geral ^20^.

Foi consenso entre os entrevistados de todos os grupos o predomínio da assistência organizada a partir de patologias na APS, com protocolos definidos, metas de acompanhamento e financiamento por programas, que condicionavam múltiplos e fragmentados contatos dos usuários com os serviços, a depender das comorbidades. Mesmo nos CESFAM, predominava a atenção programática, a despeito de serem unidades representativas do MAIS. Essa foi uma das críticas mais contundentes e um dos aspectos mais desafiadores enfrentado pela universalização da APS, destacado pela maioria dos entrevistados de todos os níveis. No marco da atenção integral, foi consenso que a universalização da APS avançou pouco nas estratégias de coordenação do cuidado e que os hospitais se encontravam “distantes da reforma”, salvo experiências piloto. As longas listas de espera seguiam sendo um problema muito relevante, sobretudo para a atenção especializada, aspecto que afetava a capacidade da APS em ofertar cuidados contínuos e integrais, mesmo no âmbito da ECICEP. Nesse sentido, considerou-se que os usuários do segmento privado se sentiam mais satisfeitos pela oportunidade de acesso.

Ainda assim, foi reconhecida a abrangência dos serviços e ações ofertados, principalmente pelos CESFAM, com uma composição ampliada de profissionais, não encontrada em outras partes do mundo em sistemas universais, tampouco nas Isapres que garantiam acesso apenas a atendimentos individuais em consultórios privados, condicionados a elevados copagamentos.

### Engajamento da comunidade e de outros atores

### Diálogos cidadãos e cartografia dos ativos comunitários

Os diálogos cidadãos, que também fazem parte dos componentes centrais da universalização da APS, expressavam o objetivo de ampliar a participação social e identificar barreiras à aceitabilidade dos serviços de APS. As Comunas Pioneiras desenvolviam um plano de ação comunal para integrar a experiência dos usuários em uma perspectiva “vinculante”. Representações comunitárias foram convidadas a apresentar e eleger projetos, que recebiam recursos da universalização da APS para a implementação.

Além disso, as Comunas Pioneiras tinham como meta obrigatória a identificação de ativos comunitários, cartografados para a composição de um mapa de recursos dispostos na plataforma de Gestão Social Local (GLS), sistema que agregava informações sobre benefícios sociais e para gestão sociossanitária nos territórios. Em conjunto com o Ministério do Desenvolvimento Social e Família foi implementado um módulo de saúde no GLS para melhorar o trabalho intersetorial e integrar os benefícios de saúde aos benefícios sociais.

Embora a ação comunitária fosse um dos motes da universalização da APS, participantes da academia destacaram um certo “cansaço” das equipes de APS, pois reconheciam não serem responsáveis por todos os determinantes sociais da saúde, sendo necessária a mobilização de outros setores das políticas sociais e atores relevantes como os prefeitos. Mesmo com a reforma, o tema dos determinantes sociais ficava “para trás” diante do predomínio do cuidado individual biomédico e excessiva ênfase no cumprimento de metas, que direcionava o trabalho das equipes. De toda forma, nos pilotos, foi relatado por representantes dos níveis meso e micro maior esforço para aproximar as ações de saúde às de desenvolvimento social. Também havia horas protegidas para a participação nos Conselhos Locais, presentes nos CESFAM, em geral, a cargo da assistente social. Ainda assim, foi enfatizado, pelo conjunto dos entrevistados, que as medidas implementadas não eram suficientes para alcançar a participação social “vinculante”. 

## Discussão

A proposta da universalização da APS representa a principal ação do governo chileno (2022-2026) de reforma do subsistema público. Embora as expressivas movimentações sociais de 2019 e a eleição de um governo progressista tenham representado confluência favorável a um redirecionamento das políticas sociais, as alternativas ou soluções que se seguiram no período pós-eleitoral parecem não ter compreendido ou contemplado os valores vigentes da maioria da sociedade ^21^. Assim, na ausência de convergência entre problemas, soluções e fluxo político, mudanças estruturais, a exemplo de um sistema de saúde universal de direito, não se tornaram viáveis. Na saúde, algumas proposições foram combinadas e recolocadas na agenda política, entre as quais a universalização da APS, que conflui apoios dos campos político, social e corporativo de distintos espectros ^21^.

A proposta da universalização da APS busca fortalecer as bases do subsistema público que, de fato, é responsável pela cobertura da maior parte da população chilena. Em acordo ao marco teórico conceitual adotado nesta análise, a implementação da universalização da APS fundamenta-se em uma abordagem integral da APS com a criação de novos espaços de governança, incremento do financiamento e componentes de melhoria do acesso ao cuidado individual, com a incorporação de tecnologias digitais e ação nos territórios, com alcance diferenciado entre os componentes. Nesse sentido, a universalização APS converge e representa um novo impulso ao processo anterior de reforma da APS a partir do MAIS. As medidas alinham-se a estratégias adotadas em países de baixa, média e alta rendas que, todavia, enfrentam desafios relacionados à retenção da força de trabalho, prestação de serviços de qualidade, engajamento comunitário, ação intersetorial, falhas nas estruturas de governança e insuficiência dos recursos disponíveis ^22^. Embora a reforma tenha alcançado bons resultados na maioria dos indicadores previstos, dificuldades relacionadas à abordagem familiar e qualidade indicam a resiliência do enfoque biomédico ^23^.

Na construção de viabilidade para a implementação da universalização da APS, em contexto de conflitos políticos e fiscais, duas estratégias se destacaram - a conformação de uma estrutura de governança e os projetos pilotos. O conselho e comissões são conformados por atores com reconhecido capital social e político, que ao favorecerem a credibilidade e a confiança ampliam a capacidade de governança ^24^. Em que pese a força de um conselho que congrega ex-ministros da saúde do governo de direita de Sebastián Piñera à esquerda (de Ricardo Lagos e Michelle Bachelet), o caminho percorrido para a implementação da universalização da APS transcorre na ausência da aprovação de um marco legal. Embora a ausência de leis fragilize a sustentabilidade e a continuidade da proposta ^2^, a avaliação que prevaleceu foi a de que o fato de o governo não ter maioria no parlamento inviabilizaria o início do projeto ou implicaria em descaracterização do desenho proposto.

Projetos piloto para a implementação de inovações, testadas em pequena escala, atendem ao propósito de avaliar a viabilidade de determinada proposição ou política de saúde ^25^. No entanto, para que ocorra a expansão de forma sustentável, os objetivos finalísticos devem estar presentes desde o início. Nesse sentido, várias recomendações devem ser consideradas desde a formulação dos pilotos ^25^, parte das quais observadas na experiência analisada, como a construção de um processo participativo envolvendo atores-chave; teste e adaptação da proposta às configurações socioculturais e institucionais dos diversos contextos; definição de um marco avaliativo; desenvolvimento de estratégias comunicacionais e de intercâmbio que tornem os pilotos uma experiência de aprendizado institucional. Por outro lado, a expansão da universalização da APS deve estar balizada pelo estabelecimento de consenso em torno da sua necessidade, convencimento de setores governamentais e não governamentais sobre os recursos financeiros para a sua sustentabilidade ao longo do tempo, e, como já sinalizado, convergência política para a defesa legislativa do projeto, pontos ainda não equacionados, no caso analisado.

Em relação aos eixos estratégicos, a inscrição universal, embora avaliada positivamente pela maior parte dos atores-chave, gera questionamentos quanto à capacidade de incorporar novas parcelas da população. De toda forma, o incentivo à inscrição logrou alcançar, principalmente, populações vinculadas ao Fonasa que não acessavam os centros de saúde ou faziam uso da modalidade de livre eleição ^9^. A incorporação das camadas de renda média da população produz um conjunto de efeitos positivos, tais como maior disponibilidade de recursos para o Fonasa, proveniente de afiliados jovens com baixa carga de doenças e uso dos serviços de saúde, que passam a contribuir com o financiamento do setor público. De todo modo, o risco de aumentar as iniquidades é uma preocupação na medida em que, segundo dados de 2020, entre os beneficiários Fonasa, 44% dos inscritos eram atendidos exclusivamente em serviços de APS, enquanto cerca de 1,5 milhão buscavam atendimento no setor privado por meio da Modalidade de Livre Eleição, seja por problemas de acesso, percepção de baixa resolutividade, entre outros ^26^. Ainda assim, no caso chileno, em que predomina uma concepção neoliberal na provisão de políticas públicas, que parece não arrefecer, ampliar o acesso à cobertura pública de APS de qualidade pode ser estratégico para angariar apoios para a expansão, financiamento e sustentabilidade da proposta de universalização do sistema público.

Dificuldades em acessar os cuidados primários fora do horário regular de funcionamento dos serviços representa uma das causas de renúncia ou dificuldades na obtenção de atenção em saúde, mais comumente vivenciadas pelos grupos de renda mais baixos e com múltiplas condições crônicas ^27^ ou população jovem trabalhadora. No Brasil, um estudo indicou que o maior número de atendimentos em horário estendido em UBS contemplou adultos entre 20 e 59 anos, sendo a maioria com demandas relacionadas a doenças crônicas não transmissíveis, como a hipertensão arterial e a diabetes ^28^.

Em cenários no quais o agendamento para atendimento na APS ocorre apenas presencialmente, com enfrentamento de filas, a possibilidade do contato remoto pode ser considerada um facilitador do acesso, sobretudo para usuários com mobilidade reduzida ou com horários de trabalho conflitantes ^29^. Simultaneamente, o manejo remoto exige reorganização dos processos de trabalho das equipes e comunicação com a população, conforme sinalizado neste estudo. De todo modo, as duas medidas - gestão remota e extensão do horário - são sinérgicas na ampliação do acesso, aliando tecnologia da informação ^26^ e possibilidade de contatar os serviços fora dos horários convencionais.

No caso chileno, a despeito da reforma MAIS, de forma geral persiste a atenção curativa fragmentada por programas verticais, fortalecida pela priorização seletiva do GES, pelo desenho do financiamento e desconhecimento do modelo pelos demais níveis assistenciais ^7^. A ECICEP busca recuperar diretrizes e estratégias do MAIS ^20^. Nesse sentido, ainda que a adesão seja incipiente, busca enfrentar a excessiva especialização, centralização na atenção hospitalar e necessidade de novas diretrizes que atendam às demandas complexas de uma população cada vez mais envelhecida em sistemas de saúde com recursos limitados, desafios presentes em outros cenários ^30^.

Parte expressiva dos entrevistados definiram a participação social na saúde como “não-vinculante”. A centralização da política de saúde no Minsal garante unidade nas ações, por outro lado, limita a participação externa a espaços formais, nem sempre de forma contínua e progressiva ^31^. Na APS municipal, a participação social é mais efetiva ^31^ e a implementação de projetos aprovados pelos usuários, com recursos da universalização da APS, pode fortalecer o engajamento ao superar um certo esgotamento da participação consultiva ^32^.

Por fim, destaca-se a convergência e complementariedade entre os grupos de atores entrevistados quanto aos êxitos e desafios na implementação da universalização da APS, destacando-se a sinergia em torno da identificação dos nós críticos. Tal característica imprimiu certa homogeneidade na descrição e análise da experiência.

## Considerações finais

O alcance de melhorias nos resultados ou no acesso aos serviços de saúde por meio de reformas ou inovações, principalmente em países de baixa e média rendas, depende da constituição de uma estrutura de governança, com o comprometimento político de indivíduos e instituições, estabilidade, memória institucional com registro e monitoramento contínuo da experiência, além de políticas relevantes e apropriadas às circunstâncias e contextos ^33^, aspectos identificados na experiência analisada. Os desafios para os próximos anos será a sustentabilidade e expansão da universalização da APS com base nas lições aprendidas, mesmo em face às turbulências políticas e econômicas que ciclicamente ameaçam a garantia do direito e acesso universal em saúde no contexto da frágil democracia que caracteriza os países da região ^14^. Especificamente, os desafios estruturais relacionados ao financiamento e à organização do subsistema público ^34^ seguirão como pautas pendentes que requerem reformas profundas.

Por fim, cabe destacar que os sistemas de saúde refletem valores políticos, institucionais, sociais e culturais de um país, o que delineia distintos caminhos. De toda forma, compartilhar experiências e inovações, sobretudo entre países latino-americanos, pode informar as políticas e ações de saúde potencialmente capazes de enfrentar as persistentes desigualdades sociais, bem como valorizar as iniciativas e grandes esforços empreendidos na construção de uma APS resiliente, equânime e customizada à diversidade que caracteriza nossos territórios e populações.

## Data Availability

As fontes de informação utilizadas no estudo estão indicadas no corpo do artigo.
